# Supplementary material for: A 10-Year Retrospective Study on Pediatric Visceral Leishmaniasis in a European Endemic Area: Diagnostic and Short-Course Therapeutic Strategies
Source: Healthcare (Basel). 2023 Dec 21;12(1):23. doi: 10.3390/healthcare12010023 (PMC10779246; doi:10.3390/healthcare12010023)
Supplement: Supplementary file 1 [file healthcare-12-00023-s001.zip › healthcare-2698532-supplementary.pdf]

## Supplementary Materials

**Table S1.** Diagnostic criteria for hemophagocytic lymphohistiocytosis (HLH), from Henter et al [23]. For the diagnosis of HLH five of the eight criteria must be fulfilled.

| **Diagnostic criteria for HLH** |
| --- |
| a. Fever (≥ 38.5 °C) |
| b. Splenomegaly |
| c. Cytopenias (affecting at least 2 of 3 lineages in the peripheral blood with hemoglobin < 10 g/dL, platelets < 100 × 109/L, and neutrophils < 1.0 × 109/L) |
| d. Hypertriglyceridemia and/or hypofibrinogenemia (fasting triglycerides ≥ 265 mg/dL, fibrinogen < 150 mg/dL) |
| e. Hemophagocytosis in bone marrow or spleen or lymph nodes and no evidence of malignancy |
| f. Low or absent NK cell activity (according to local laboratory) |
| g. Ferritin > 500 μg/L |
| h. Soluble CD25 (interleukin-2 receptor) ≥ 2400 U/mL |

NK: natural killer.

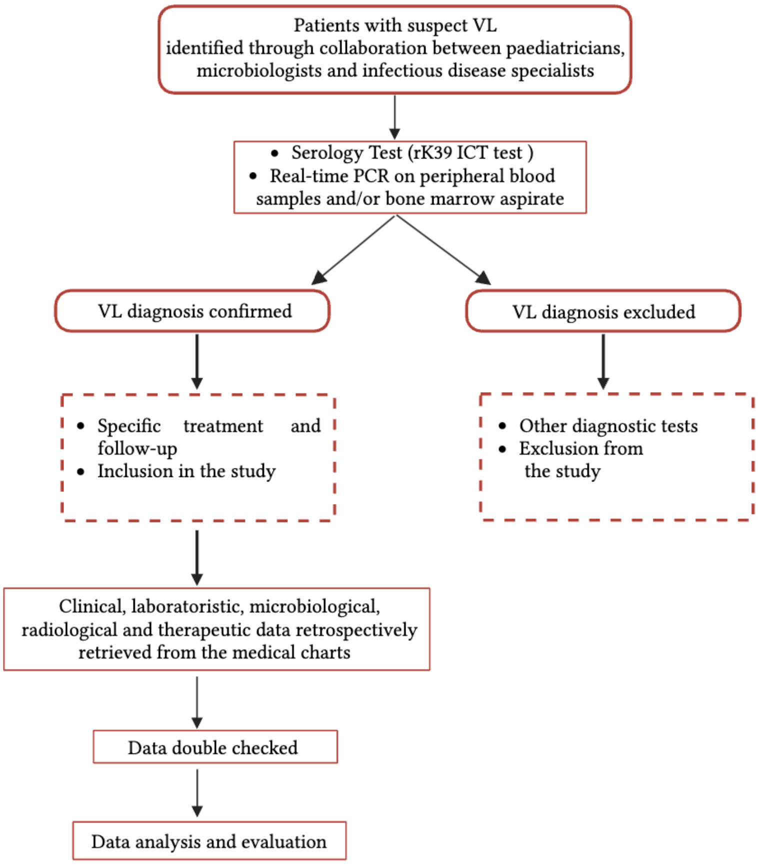


**Figure S1.** Flowchart of the study procedure. VL: visceral leishmaniasis.
